# Legacy and Emerging Contaminants in Demersal Fish Species from Southern Norway and Implications for Food Safety

**DOI:** 10.3390/foods9081108

**Published:** 2020-08-12

**Authors:** Marco Parolini, Sara Panseri, Federico Håland Gaeta, Federica Ceriani, Beatrice De Felice, Maria Nobile, Giacomo Mosconi, Trond Rafoss, Francesco Arioli, Luca Maria Chiesa

**Affiliations:** 1 Department of Environmental Science and Policy, University of Milan, via Celoria 26, I-20133 Milan, Italy; beatrice.defelice@unimi.it; 2 Department of Health, Animal Science and Food Safety, University of Milan, Via Celoria 10, I-20133 Milan, Italy; sara.panseri@unimi.it (S.P.); federica.ceriani@unimi.it (F.C.); maria.nobile1@unimi.it (M.N.); giacomo.mosconi@unimi.it (G.M.); luca.chiesa@unimi.it (L.M.C.); 3 Norwegian Institute for Water Research (NIVA), N-4879 Grimstad, Norway; Federico.Gaeta@niva.no; 4 Department of Natural Sciences, University of Agder (Uia), 4630 Kristiansand, Norway; trond.rafoss@uia.no

**Keywords:** food safety, organohalogen compounds, fish

## Abstract

The present study aimed at measuring the levels of legacy and emerging contaminants in fillet samples from four demersal fish caught in two fishing sites from Southern Norway, in order to assess possible implications for food safety. Levels of organochlorine compounds (OCs), organophosphate pesticides (OPs), polychlorinated biphenyls (PCBs), polybromodiphenyl ethers (PBDE), per- and polyfluoroalkyl substances (PFASs), and polycyclic aromatic hydrocarbons (PAHs) were measured in fillet from Atlantic cod (*Gadus morhua*), European plaice (*Pleuronectes platessa*), lemon sole (*Microstomus kitt*), and European flounder (*Platichthys flesus*) specimens. A negligible contamination by all the investigated chemicals was noted in both the fishing sites, as very low levels of OCs, PCBs, and PFASs were noted in a limited number of individuals for each species. Considering the levels of contaminants measured in fillets of the four demersal fish species, negligible risk for human health for Norwegian consumers can be supposed.

## 1. Introduction

The release of both legacy and emerging contaminants represents a serious concern for marine ecosystems. The presence and distribution of persistent organic pollutants (POPs), such as polychlorinated biphenyls (PCBs), organochlorine compounds (OCs) and organophosphate pesticides (OPs), as well as polybrominated diphenyl ethers (PBDEs), per- and polyfluoroalkyl substances (PFASs), and polycyclic aromatic hydrocarbons (PAHs), is considered an alarming environmental threat because of their persistence, capability to bioaccumulate, and toxicity [1]. All these organic contaminants can be accumulated over the trophic chain, resulting in high concentrations and more dangerous adverse effects towards top predators, including humans [1,2,3]. For these reasons, there is an urgent need for understanding the levels, the trends, and the impacts due to the bioaccumulation of these contaminants in marine food webs, to ultimately estimate the potential threat for human health. Fish species play a pivotal ecological role in aquatic ecosystems and can accumulate to a great extent in a great number of lipophilic contaminants [4]. For this reason, fish species are commonly used to biomonitor the levels of organic contaminants worldwide and the potential implication for human health [5,6,7]. Although several monitoring surveys have demonstrated that the levels of some groups of halogenated compounds, such as PCBs and PBDEs, are declining in the marine ecosystems during the last decades [8,9,10], notable concentrations of these contaminants are still measured in fish from various geographical areas [10,11,12,13,14]. In Europe, the contamination by organohalogen compounds has been mainly monitored in diverse pelagic fish species from the Mediterranean Sea [15,16,17,18] or northern sea areas [13,19,20]. However, the information on the levels and trends of POPs in fish from Norwegian seawaters is limited and predominantly referred to contamination of commercial fish species from high-latitudes seas [21,22] or coastal and fjord areas [23]. To date, no study has investigated the contamination of fish collected in one of the more polluted fjords in Norway, namely the Flekkefjord fjord [24]. Industrial activities and municipal waste contributed to the contamination of this fjord, as documented by a series of reports pointing out notable levels of PCBs and heavy metals in seawater, sediments, and biota from various locations within the fjord [25,26]. Moreover, a recent six-month active biomonitoring study using transplanted blue mussels (*Mytilus edulis* sp.) highlighted an increase in the fjord contamination by PCBs and PAHs as a consequence of a huge undersea landslide, which provoked the re-suspension of contaminated sediments [27]. The results from this survey suggested a potential risk for mussel predators and human health because the levels of PCBs and PAHs exceeded the limits set by the European Commission for the consumption of mollusks [27]. According to these findings, fish species living in the Flekkefjord fjord might accumulate high levels of PCBs, PAHs, and other organic contaminants. This is particularly true for demersal fish species feeding on benthic organisms and living in close contact with sea bottom sediments, whereby the levels of contamination are higher compared to the upper layers of the water column [27]. In fact, our previous biomonitoring study performed in five locations close to the fishing area of Lafjorden demonstrated that blue mussels caged at a 15 m depth accumulated higher levels of PCBs and PAHs compared to conspecifics caged at a 5 m depth [27]. As fishery products are considered as one of the main contributors to the intake of organic contaminants in the human diet [28], the consumption of fish living in the Flekkefjord fjord might represent a risk for the population consuming fish from this specific area. Thus, the present study was aimed at measuring the concentrations of legacy (i.e., organochlorine compounds—OCs, organophosphate pesticides—OPs, polychlorinated biphenyls—PCBs, and polycyclic aromatic hydrocarbons—PAHs) and emerging contaminants (i.e., polybromodiphenyl ethers—PBDEs and per- and polyfluoroalkyl substances—PFASs), in the fillets of four demersal fish species collected in two fishing areas from Southern Norway near the Flekkefjord fjord. The levels of contaminants were measured in fillets from four fish species belonging to different trophic levels commonly fished and consumed by the Norwegian population, namely Atlantic cod (*Gadus morhua*), lemon sole (*Microstomus kitt*), European plaice (*Pleuronectes platessa*), and European flounder (*Platichthys flesus*). The final goal of this study was to investigate the potential risk for fish consumers’ health by comparing the levels of investigated contaminants with consumer safety thresholds.

## 2. Materials and Methods

### 2.1. Fish Sampling

**Figure 1:**
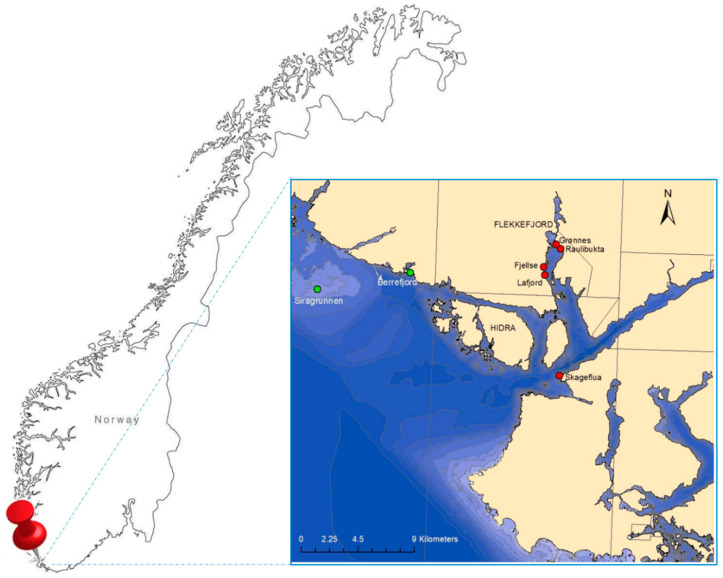
Geographical localization of the fishing site of Siragrunnen (green dots) and Lafjorden (red dots) in Southern Norway.

A total of 65 fish belonging to four demersal species were caught, through nets, by a professional fisherman between January the 30th and March the 26th 2019 in two different fishing sites form Southern Norway near the Flekkefjord fjord (Figure 1). In detail, fish were caught at Lafjorden (58°15.867′, 6°38.919′) and neighboring locations (i.e., Fjellse, Grønnes, Skageflua, and Raulibukta), which was located within the Flekkefjord fjord near the city of Flekkefjord, where the re-suspension of contaminated sediments occurred in 2018 [27]. The second fishing site was the open-sea shoal named Siragrunnen (58°14.899′, 6°20.500′) and its close location Berrefjords, which should not suffer from contamination coming from current human activities and re-suspension of contaminated sediments from the Flekkefjord fjord. In the open-sea shoal of Siragrunnen, the fishermen put the nets in the water to fish at ca. 30 m depths, while in Lafjorden, the nets were lowered at depths ranging between 15 and 30 m depending on the depth of the sea bottom. At Lafjorden, nets were lowered at depths similar to those where cages containing blue mussels were transplanted in five locations within the Flekkefjord fjord [27], not so far from the fishing area of Lafjorden. Thus, we can reasonably state that demersal fish sampled in the Flekkefjord fjord (i.e., Lafjorden) experienced similar exposure compared to blue mussels. Three fish species were collected at both the sampling sites, namely Atlantic cod (*Gadus morhua*), lemon sole (*Microstomus kitt*), and European plaice (*Pleuronectes platessa*), while specimens of European flounder (*Platichthys flesus*) were fished at Lafjorden only. The four species of demersal fish considered in this study have a different diet and belong to dissimilar trophic levels. Atlantic cod is a top predator active hunter, mainly preying on herrings and sprats. The lemon sole lives preferentially on stony bottoms and feeds on a variety of small invertebrates, mainly polychaetes. The European plaice and the European flounder both live on mud and the sand bottom in the sea and estuaries but the first species feeds mainly on thin-shelled mollusks and polychaetes, while the second one feeds on benthic fauna, including small fishes and invertebrates (Available online: http://www.fishbase.in; accessed on 06 August 2020). Thus, the lemon sole, the European plaice, and the European flounder belong to a lower level of the trophic chain compared to the Atlantic cod.

In detail, at Siragrunnen, 14 Atlantic cods (6 males and 8 females), 5 lemon soles (2 male and 3 females), and 4 European plaices (2 males and 2 females) were fished (Table S1). At Lafjorden, 18 Atlantic cods (10 males and 8 females), 11 European flounders (5 males and 6 females), 7 lemon soles (1 male, 4 females, and 2 immatures), and 6 European plaices (2 males and 4 females) were fished (Table S2). Fish were quickly transported in iceboxes to the fish market of Flekkefjord, where they were measured (i.e., total length) and weighed (Table S1). Subsequently, a little portion of the fillet (about 3 g) below the dorsal fin was collected from each fish and stored at −20 °C until chemical analyses.

### 2.2. Chemicals and Reagents

Standard solutions of organochlorine pesticides (OCs) and their metabolites, as well as of organophosphate pesticides (Ops) (see Table 1 for the list of OCs and OPs), were purchased from Restek (Bellefonte, PA, USA). Similarly, standard solutions of polycyclic aromatic hydrocarbons (i.e., chrysene, benzo(α)anthracene, benzo(β)fluoranthene, and benzo(α)pyrene) were purchased from Restek (Bellefonte, PA, USA). Mixed solutions of PCB congeners (see Table 2 for the list) and CB-209, which was used as an internal standard (IS) for PCBs and PAHs, as well as mixed solutions of PBDE congeners (BDE-28; BDE-33; BDE-47; BDE-99; BDE-100; BDE-153; and BDE-154) and fluorobromodiphenyl ether (FBDE), which was used as IS for flame retardants, were purchased from AccuStandard (New Haven, CT, USA). The standard solutions of per- and polyfluoroalkyl substances (PFASs; see Table 3 for the list), as well as the two 13C-labeled IS of MPFNA and MPFOS, were purchased from AccuStandard (New Haven, USA). The QuEChERS ‘SupelTM QuE Citrate (EN) tubes for extraction and SupelTM QuE-ZSEP (EN) tubes for the clean-up step used for analyses of OCs, OPs, PCBs, PAHs, and PBDEs were purchased from Supelco (Sigma-Aldrich, St. Louis, MO, USA). The extraction cartridges used for PFASs analyses (Oasis WAX 3 mL, 60 mg) were provided by Waters (Milford, MA, USA).

### 2.3. Analysis of Chlorinated and Brominated Compounds and PAHs

The extraction of chlorinated and brominated compounds (OCs, OPs, PCBs, and PBDEs), as well as PAHs, from the fish fillet was performed by means of the QuEChERS approach as described elsewhere [29]. An aliquot of about 3 g of fish fillet was homogenized, transferred to a QuEChERS extraction tube, and ISs were added. After the addition of a mixture (4:1 *v*/*v*) of hexane/acetone (10 mL) and shaking, the sample was centrifuged for 10 min at 5000× *g* at 4 °C. Then, the supernatant was transferred to a QuEChERS extraction tube, shaken, and centrifuged as described above. The supernatant was transferred to a clean-up tube (Z-Sep) to remove interferences. After evaporation (at 35 °C in a centrifugal evaporator), the residual sample was dissolved in hexane (1 mL) and injected to a GC/MS-MS for quali-quantification of contaminants. Triple quadrupole mass spectrometry (QqQ) in electronic impact (EI) mode was used to simultaneously detect and quantify contaminants, according to the conditions described in our previous work [30]. A GC Trace 1310 chromatograph coupled to a TSQ8000 triple quadrupole mass detector (Thermo Fisher Scientific, Palo Alto, CA, USA) was used to confirm and quantify contaminants using a fused-silica capillary column Rt-5MS Crossbond-5% diphenyl 95% dimethylpolysiloxane (35 m × 0.25 mm i.d., 0.25 μm film thickness, Restek, Bellefonte, PA, USA). The QqQ mass spectrometer was operated in selected reaction monitoring mode (SRM), detecting two-three transitions per analyte. The identification of chlorinated and brominated compounds and PAHs was performed through the comparison of the peak of each compound at a specific retention time with that obtained from the same compound in the standard solution, as well as by comparing the MS/MS fragmentation spectra obtained for each compound. A calibration solution of mixed compounds was prepared daily through serial dilution of stock solutions (10 µg/mL in hexane) and the proper volume was used as a spiking solution. The XcaliburTM processing and instrument control software program and Trace Finder 3.0 for data analysis and reporting (Thermo Fisher Scientific) were used. The limit of detection (LOD), limit of quantification (LOQ), repeatability (coefficient of variation—CV%), and recovery (%) for each investigated compound are reported in Table S3.

### 2.4. Analysis of Per- and Polyfluoroalkyl Substances (PFASs)

The analysis of per- and polyfluoroalkyl substances in the fish fillet was performed as described elsewhere [7,30]. Briefly, ~3 g of sample were spiked with the ISs at the concentration of 5 ng/mL and 10 mL of acetonitrile were added for extraction and protein precipitation. After vortexing and 15 min of sonication, the sample was centrifuged (2500×*g* at 4 °C for 10 min), and the supernatant was evaporated (at 40 °C in a rotary vacuum evaporator). The extract was resuspended in water (10 mL) and purified under vacuum filtration on SPE Oasis WAX Cartridges, previously conditioned with 0.5% ammonium hydroxide in methanol, methanol, and Milli-Q water (3 mL for each solution). Then, cartridges were washed with 25 mM acetate buffer (3 mL, pH 4.5) and 2 mL of methanol. The elution was performed with 3 mL of ammonium hydroxide (0.5% in methanol). The eluate was dried and suspended in 100 μL of methanol:ammonium formate (20 mM; 10:90 *v*/*v*). The quali-quantification of PFAS was performed in an HPLC system (Thermo Fisher Scientific, San Jose, CA, USA) coupled to a QExactive Orbitrap (Thermo Scientific, San Jose, CA, USA). The instrument was equipped with a heated electrospray ionization (HESI) source operating in negative mode. A Synergi Hydro-RP reverse-phase HPLC column (150 × 2.0 mm, 4 μm particle size), with a C18 guard column (4 × 3.0 mm) (Phenomenex, Torrance, CA, USA), was used for the chromatographic separation. To minimize background contamination, we used stainless steel capillary tubes. Because of the presence of PFOA and PFOS in the chromatographic system, a small Megabond WR C18 column (5 cm × 4.6 mm, i.d. 10 μm) was included between the pump and the injector. This column allowed the delay of the elution of compounds by 2 min compared to the analytes present in the samples. Aqueous ammonium formate (solvent A, 20 mM) and methanol (solvent B) were used as mobile phases. The gradient and the mass parameters are described elsewhere [30]. Xcalibur^TM^ 3.0 software (Thermo Fisher Scientific, San Jose, CA, USA) was used to control the HPLC-HRMS system and to elaborate the data. Working solutions (10 and 100 ng/mL in methanol) were prepared through serial dilutions of the stock solutions (1 mg/mL) during each analytical session. The limit of detection (LOD), limit of quantification (LOQ), repeatability (coefficient of variation—CV%), and recovery (%) for each investigated PFAS are reported in Table S3.

### 2.5. Quality Assurance and Quality Control

The methods were checked for their linearity, repeatability, recovery, limit of detection (LOD), and quantification (LOQ), as reported in Table S3 according to the SANTE 2017 [31] guidelines and our previous studies [7,29,30]. The LOD and LOQ for each analyzed compound were calculated from the calibration curve in the concentration range corresponding to the lower concentration levels according to MRL for each compound, when available. LOD was calculated according to the equation LOD = 3.3 SD0/slope, where SD0 corresponds to the residual standard deviation. LOQ was calculated as 3 × LOD. Recovery for each contaminant was performed at a fortification level of 5 ng/g for chlorinated and brominated compounds, as well as for PAHs, while at a fortification level of 50 pg/g for PFASs. The repeatability of the methods (expressed as the coefficient of variation, CV%) was assessed through the analysis of six replicates by spiking a known amount of standard solution (5 ng/g for OCs, OPs, PBDEs, and PAHs and 50 pg/g for PFASs) during fish homogenization.

## 3. Results and Discussion

**Figure 2:**
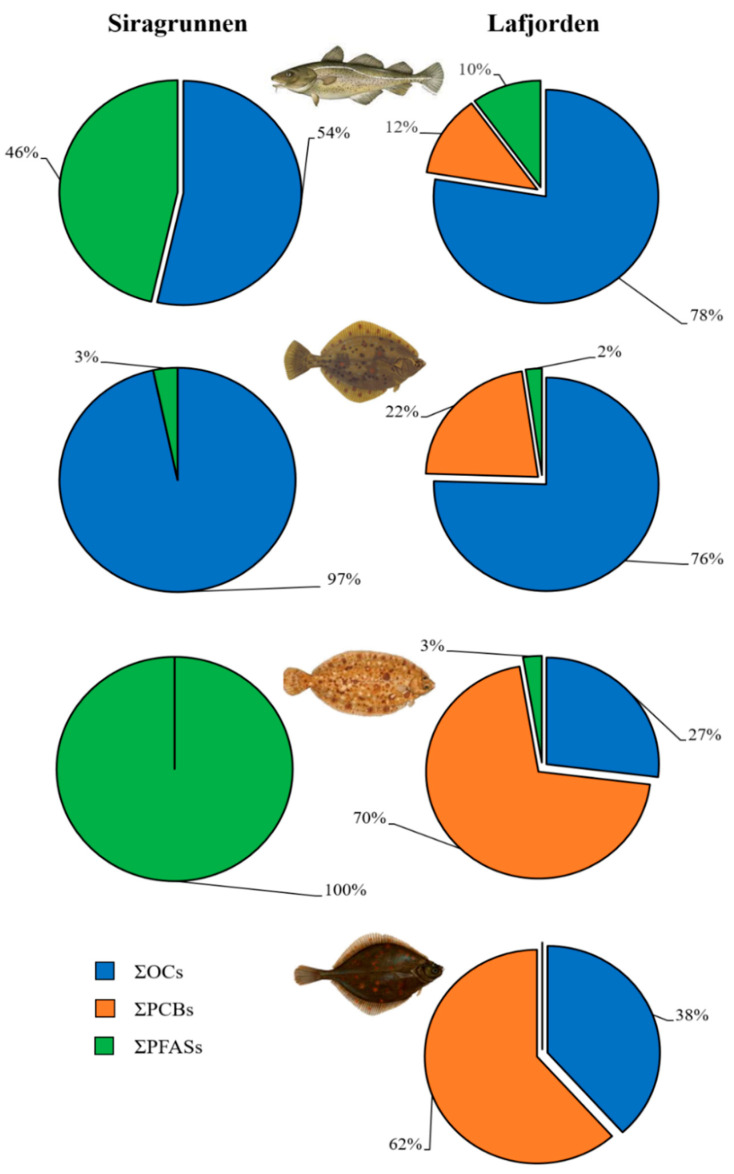
Percentage contribution (%) of organohalogen contaminants in the fillets of four demersal fish species (from the top of the picture: Atlantic cod, European flounder, lemon sole, and European plaice) caught at Siragrunnen (open-sea shoal) and Lafjorden (within Flekkefjord fjord). To calculate the mean for each compound, according to EFSA (2010), we used the LOD value when the level of a specific compound was <LOQ.

Our results showed that the contamination by halogenated compounds and PAHs in fillets from four demersal fish species caught in Southern Norway was negligible. In detail, OPs and PBDEs were not detected in the fillet of fish from both the fishing sites (data not shown). These results agreed with those from a previous study reporting that no OPs and PBDEs were accumulated in soft tissues of blue mussels caged in five locations within the Flekkefjord fjord over a six-month period in 2018 [27]. No PAHs were detected in fish from Lafjorden and from Siragrunnen (data not shown) despite a notable increase of these contaminants, mainly benzo(α)pyrene, being found in blue mussels caged in Flekkefjord fjord during the 2018 survey [27]. The OCs contamination was negligible both in fish from Siragrunnen and Lafjorden (Table 1), in accordance with levels measured in blue mussels from Flekkefjoerd fjord [27]. Overall, detectable levels of some OCs, namely p,p′-DDT homologues, hexachlorobenzene, aldrin, heptachlor epoxide, and endosulfan II, were found in 23% of the fish samples and, specifically, in 29% of the fish collected at Lafjorden and 13% of the fish from Siragrunnen. However, the levels of detected OCs were below the limit of quantification (<LOQ), with the exception of the o,p′-DDT concentration measured in the fillet from a European flounder individual caught at Lafjorden (5.73 ng/g fresh weight). Overall, PCBs (Table 2) were detected only in 28% of the fish, mainly in those from Lafjorden (40%). However, in most of the fish, the levels of PCB congeners were <LOQ, pointing out a negligible PCB contamination in all the species. However, measurable levels of PCBs were found only in 5% of the fish and, specifically, in the fillet of a European flounder (ΣPCBs = 3.55 ng/g fresh weight), a lemon sole (ΣPCBs = 4.63 ng/g fresh weight), and a European plaice (ΣPCBs = 13.7 ng/g fresh weight) caught at Lafjorden. The PCB levels measured in demersal fish species from Southern Norway were similar to those measured in halibut (*Hippoglossus hippoglossus*) from coastal areas of the far north of Norway (median concentration = 2.8 ng/g lipid weight) [21]. The CB-138 was detected in all three fish species, representing the main congener characterizing the PCB fingerprint, while CB-180 was measured in the fillet from European flounder only, accounting for 39% of the fingerprint for that specific fish. The low amount of PCBs measured in fish from Lafjorden might come from the Flekkefjord fjord, where, in 2018, a notable increase of these contaminants was recorded in blue mussels as a consequence of the re-suspension of contaminated sediments due to an undersea landslide [27]. Thus, even though the PCB concentrations measured in all the fish species form Lafjorden were very low and often <LOQ, they might be reasonable due to the re-suspension and the subsequent dispersion of PCB-contaminated sediments from the Flekkefjord fjord [27]. These results suggest that PCBs measured in blue mussels caged in five locations within the Flekkefjord fjord can be transmitted to higher trophic levels of this ecosystem. This hypothesis was supported by the lack of PCBs measured in fish from the open-sea shoal at Siragrunnen, which did not suffer contamination from the Flekkefjord fjord, as well as by the analysis of the contamination fingerprint of fish fillets. In fact, the detection frequency and the concentrations of contaminants were generally higher in fish from Lafjorden than those from Siragrunnen (Table 4). Moreover, the fingerprint of the contamination also differed between the fishing areas (Figure 2). Whilst the fingerprint of fish species from Siragrunnen was dominated by OCs and PFASs, in fish from Lafjorden, PCBs were the main contributors to the contamination. Thus, differences in the contamination fingerprint suggest that fish experienced an exposure to different contaminants due to the presence of local sources of contamination. Interestingly, whilst the OCs contamination is more important when growing along the food web, a higher prevalence of PCBs in fish from Lafjorden living in strict contact with the sea bottom and feeding with benthic invertebrates (i.e., European flounder, European plaice, and lemon sole) compared to the Atlantic cod was noted. These results should indicate that PCBs were more present in lower levels of the trophic chain, suggesting the beginning of a PCB contamination in higher ones. Alternatively, differences in the PCB accumulation prevalence and contamination fingerprint could be related to the age of the fish. In fact, although we caught only adult fish, they reasonably differed in age both within the same species, among species, and between fishing areas. Despite these findings, considering the prevalence of PCBs in fish fillets from Lafjorden, the monitoring of the presence of dioxin-like PCBs, dioxins, and furans should be a priority of further studies in order to return a complete overview concerning the implication for food safety.

Interestingly, in contrast to other contaminants, PFASs were detected in measurable concentrations in all the fish species, both from Lafjorden and Siragrunnen (Table 3). In detail, measurable concentrations of PFOS only were found in 17% of the fish caught at Lafjorden and, specifically, four Atlantic cod specimens (0.22–0.38 ng/g fresh weight), a European flounder (0.12 ng/g fresh weight), and two lemon sole individuals (0.20–0.22 ng/g fresh weight). In contrast, only PFOA was detected in all four fish species caught at Siragrunnen (0.12–2.34 ng/g fresh weight). Low concentrations accompanied by frequent non-detects of PFAS measured in fish from Southern Norway were in the same range of those measured in Atlantic cod (0.29–0.34 ng/g ww), saithe (*Pollachius virens*; 0.46–0.50 ng/g ww), and Atlantic salmon (*Salmo salar;* 0.21–0.35 ng/g ww) from the Farøe Islands [32], as well as in halibut from the far north of Norway (mean concentration < 1 ng/g ww) [21]. However, ΣPFAS was lower than those measured in smoked halibut fillet from Greenland (median concentration = 8.4 ng/g ww) [33] and in Atlantic salmon (median concentration = 2.2 ng/g ww) and whitefish (median concentration = 2.2 ng/g ww) from Baltic Sea [34].

### Human Exposure

The consumption of local fishery products is considered the main exposure pathway to environmental contaminants, particularly persistent and bioaccumulative ones, and can represent a potential risk for human health [7,29,35]. The extremely low levels of contamination in fish caught in Southern Norway might be due to the homogeneously low weights and sizes due to the young age of the examined fish as compared with data on FishBase [36] and suggest no potential implications for food safety. In fact, only OCs, PCBs, and PFASs were sporadically detected in fillets from demersal fish from Lafjorden and Siragrunnen at levels below the safety thresholds set by the European regulation. To date, in spite of the growing awareness concerning the toxic effects of POPs towards human health, the European regulation set maximum levels in edible fish only for the six marker PCBs (i.e., PCB_6_), which were identified as suitable indicators for human exposure to these chlorinated compounds [37,38]. In contrast, the concentrations of OCs, as well as of other organohalogen compounds, have not been regulated yet. The EC Regulation 1259/2011 set the limit for PCB_6_ to 75 ng/g wet weight for fish and seafood. The levels of PCB_6_ measured in all the fish caught at Lafjorden and Siragrunnen were below the EU regulation, confirming the results from recent food advice for Norwegian fish consumption reporting that the current concentrations of PCBs and dioxins measured in Norwegian fish are not a cause for concern [39]. In addition, the risk for human health due to the ingestion of contaminated fish can be assessed through the calculation of the dietary exposure (DE). The DE for contaminants detected in measurable concentrations in fish from Southern Norway and for which threshold levels are currently available, namely PCBs and PFASs, was calculated as follows: DE = (Cm × IRd)/BW [40], whereby Cm reflects the concentration of contaminants measured in fish fillet (expressed as ng/g fresh weight), IRd is the mean daily ingestion rate (0.05 g/capita/day) estimated by FAOSTAT for the consumption of fish by the Norwegian population [41], and BW is the mean body weight for adults (i.e., 70 kg). The DE (range = 0.003–0.01 ng/kg body weight/day) calculated for PCB_6_ did not exceed the provisional tolerable daily intake in any of the fish samples, indicating a negligible risk for consumers of fillets from demersal fish species caught in Southern Norway. Accordingly, the DE calculated for PFOS (<0.0002 ng/kg body weight/day in all the fish) and PFOA (<0.0017 ng/kg body weight/day in all the fish) detected in the fillets of demersal fish from Lafjorden and Siragrunnen were below the EFSA guidelines for PFOS and PFOA intake, recently estimated after epidemiological studies considering new end-points. The new tolerable weekly intake (TWI) is 6 ng/kg body weight/week for PFOS and 13 ng/kg body weight/week for PFOA, corresponding, on a daily basis, to 0.86 and 1.86 ng/kg body weight/day [42]. Moreover, according to the higher concentration of PFOS detected in fish from Lafjorden (0.38 ng/kg fresh weight), the estimated weekly exposure, calculated as the daily exposure reported above and parametrized to a week, accounted for 0.0014 ng/kg body weight/day, a lower value compared to the TWI of 13 ng/kg body weight/day set by EFSA [43]. Similarly, also considering the higher level of PFOA measured in the fillet of a lemon sole from Siragrunnen (2.34 ng/g fresh weight), the estimated weekly exposure (0.0119 ng/kg body weight/day) was lower than the TWI of 6 ng/kg body weight/week for PFOA [42]. Lastly, also considering a mean consumption of 20 g/capita/day of ‘fatty’ fish among the coastal habitants of Norway [43], the TWI estimated for PFOS and PFOA remains well below the threshold levels set by EFSA.

## 4. Conclusions

The present study showed that levels of organohalogen compounds, namely OCs, OPs, PCBs, PBDEs, and PFASs, as well as of PAHs, were negligible in individuals belonging to four demersal fish species caught in two fishing sites from Southern Norway and commonly consumed by the Norwegian population. Whilst the low levels measured in the fillet of fish caught at Siragrunnen were expected, as it was an open-sea shoal that was chosen as a reference site, we expected higher levels of contaminants in fish from Lafjorden, a location close to the Flekkefjord fjord, where previous biomonitoring analyses pointed out a hazardous contamination by PCBs and PAHs. Such low levels might be due to the low duration of the exposure or a limited feeding on contaminated prey, limiting the uptake of contaminants from the Flekkefjord fjord. Another possible reason was related to the age of fish. Although we measured contaminants in fillets from adult fish, we did not age each specific individual. As the accumulation of contaminants is affected by the individual age, we might suppose that using older fish should return higher levels of contaminants. Then, we investigated the levels of contaminants in demersal fish species with different diets and feed habits compared to pelagic top predators, which are the fish with higher levels of accumulated contaminants.

Additionally, considering the higher concentrations of compounds detected in fish fillets of the four species, namely PCBs and PFASs, the measured levels were too low to exceed the benchmark doses set by the European regulation for food safety assessment, suggesting no risk for the consumer. However, further monitoring surveys focusing on bigger or older individuals of the four demersal fish species we considered in the present study should be a priority in order to estimate an accurate human dietary exposure and to shed light on the potential risk for the consumption of fishery products in Southern Norway.

## Figures and Tables

**Table 1:** Levels of organochlorine compounds (OCs) measured in the fillet of four demersal fish species caught at Siragrunnen and Lafjorden. n.d. = not detected (i.e., <LOD = below the limit of detection); <LOQ = below the limit of quantification. LOD for all the OCs = 1.5 ng/g fresh weight; LOQ for all the OCs = 5.0 ng/g fresh weight. Levels are expressed as ng/g fresh weight.

Id	Species	α-HCH	β-HCH	γ-HCH	HCB	Heptachlor	Aldrin	Heptachlor Epoxide	Trans Chlordane	Endosulfan I	Endosulfan II	Endosulfan Sulfate	Endrin	o,p′-DDT	p,p′-DDD	p,p′-DDE
**Siragrunnen**																
OS 1/19-14/19	Atlantic cod	n.d.	n.d.	n.d.	n.d.	n.d.	n.d.	n.d.	n.d.	n.d.	n.d.	n.d.	n.d.	n.d.	n.d.	n.d.
OS 100/19–104/19	Lemon sole	n.d.	n.d.	n.d.	n.d.	n.d.	n.d.	n.d.	n.d.	n.d.	n.d.	n.d.	n.d.	n.d.	n.d.	n.d.
OS 105/19	European plaice	n.d.	n.d.	n.d.	n.d.	n.d.	n.d.	n.d.	n.d.	n.d.	n.d.	n.d.	n.d.	n.d.	n.d.	n.d.
OS 106/19	European plaice	n.d.	n.d.	n.d.	n.d.	n.d.	n.d.	n.d.	n.d.	n.d.	n.d.	n.d.	n.d.	n.d.	n.d.	<LOQ
OS 107/19	European plaice	n.d.	n.d.	n.d.	<LOQ	n.d.	<LOQ	n.d.	n.d.	n.d.	n.d.	n.d.	n.d.	n.d.	n.d.	<LOQ
OS 108/19	European plaice	n.d.	n.d.	n.d.	<LOQ	n.d.	n.d.	n.d.	n.d.	n.d.	<LOQ	n.d.	n.d.	n.d.	n.d.	<LOQ
**Lafjorden**																
La 3/19	Atlantic cod	n.d.	n.d.	n.d.	n.d.	n.d.	n.d.	<LOQ	n.d.	n.d.	n.d.	n.d.	n.d.	n.d.	n.d.	<LOQ
La 4/19	Atlantic cod	n.d.	n.d.	n.d.	n.d.	n.d.	n.d.	n.d.	n.d.	n.d.	n.d.	n.d.	n.d.	n.d.	n.d.	<LOQ
La 5/19	Atlantic cod	n.d.	n.d.	n.d.	n.d.	n.d.	n.d.	n.d.	n.d.	n.d.	n.d.	n.d.	n.d.	n.d.	<LOQ	<LOQ
La 15/19	Atlantic cod	n.d.	n.d.	n.d.	<LOQ	n.d.	n.d.	n.d.	n.d.	n.d.	n.d.	n.d.	n.d.	n.d.	n.d.	n.d.
La 6/19–14/19; La 16/19–19/19; La 1/19; La 2/19;	Atlantic cod	n.d.	n.d.	n.d.	n.d.	n.d.	n.d.	n.d.	n.d.	n.d.	n.d.	n.d.	n.d.	n.d.	n.d.	n.d.
La 103/19; La 104/19; La 106/19; La 108/19; La 110/19; La 115/19; La 116/19; La 122/19	European flounder	n.d.	n.d.	n.d.	n.d.	n.d.	n.d.	n.d.	n.d.	n.d.	n.d.	n.d.	n.d.	n.d.	n.d.	n.d.
La 105/19	European flounder	n.d.	n.d.	n.d.	n.d.	n.d.	n.d.	n.d.	n.d.	n.d.	n.d.	n.d.	n.d.	5.73	n.d.	<LOQ
La 109/19	European flounder	n.d.	n.d.	n.d.	n.d.	n.d.	n.d.	n.d.	n.d.	n.d.	n.d.	n.d.	n.d.	n.d.	n.d.	<LOQ
La 113/19	European flounder	n.d.	n.d.	n.d.	<LOQ	n.d.	n.d.	n.d.	n.d.	n.d.	n.d.	n.d.	n.d.	n.d.	n.d.	<LOQ
La 112/19	Lemon sole	n.d.	n.d.	n.d.	<LOQ	n.d.	n.d.	n.d.	n.d.	n.d.	n.d.	n.d.	n.d.	n.d.	n.d.	<LOQ
La 121/19	Lemon sole	n.d.	n.d.	n.d.	n.d.	n.d.	n.d.	n.d.	n.d.	n.d.	n.d.	n.d.	n.d.	n.d.	n.d.	<LOQ
La 124/19	Lemon sole	n.d.	n.d.	n.d.	n.d.	n.d.	n.d.	n.d.	n.d.	n.d.	<LOQ	n.d.	n.d.	n.d.	n.d.	<LOQ
La 102/19; La 120/19; La 125/19; La 126/19	Lemon sole	n.d.	n.d.	n.d.	n.d.	n.d.	n.d.	n.d.	n.d.	n.d.	n.d.	n.d.	n.d.	n.d.	n.d.	n.d.
La 117/19	European plaice	n.d.	n.d.	n.d.	<LOQ	n.d.	n.d.	n.d.	n.d.	n.d.	<LOQ	n.d.	n.d.	n.d.	n.d.	<LOQ
La 123/19	European plaice	n.d.	n.d.	n.d.	n.d.	n.d.	n.d.	n.d.	n.d.	n.d.	n.d.	n.d.	n.d.	<LOQ	<LOQ	<LOQ
La 107/19; La 118/19 La 119/19; La 114/19	European plaice	n.d.	n.d.	n.d.	n.d.	n.d.	n.d.	n.d.	n.d.	n.d.	n.d.	n.d.	n.d.	n.d.	n.d.	n.d.

**Table 2:** Levels of polychlorinated biphenyls (PCBs) measured in the fillet of four demersal fish species caught at Siragrunnen and Lafjorden. n.d. = not detected (i.e., <LOD = below the limit of detection); <LOQ = below the limit of quantification. LOD for all the PCBs = 0.15 ng/g fresh weight; LOQ for all the PCBs = 0.5 ng/g fresh weight. Levels are expressed as ng/g fresh weight.

Id	Species	PCB-28	PCB-52	PCB-101	PCB-138	PCB-153	PCB-180
**Siragrunnen**							
OS 1/19–11/19 OS 13/19–14/19	Atlantic cod	n.d.	n.d.	n.d.	n.d.	n.d.	n.d.
OS 12/19	Atlantic cod	n.d.	n.d.	n.d.	<LOQ	n.d.	<LOQ
OS 100/19–104/19	Lemon sole	n.d.	n.d.	n.d.	n.d.	n.d.	n.d.
OS 105/19–108/19	European plaice	n.d.	n.d.	n.d.	n.d.	n.d.	n.d.
**Lafjorden**							
La 1/19; La 2/19; La 4/19; La 5/19; La 12/19; La 16/19–18/19	Atlantic cod	n.d.	n.d.	n.d.	n.d.	n.d.	n.d.
La 3/19	Atlantic cod	n.d.	n.d.	n.d.	<LOQ	<LOQ	<LOQ
La 6/19	Atlantic cod	n.d.	n.d.	n.d.	n.d.	n.d.	<LOQ
La 7/19	Atlantic cod	n.d.	<LOQ	n.d.	n.d.	n.d.	<LOQ
La 8/19	Atlantic cod	n.d.	n.d.	n.d.	<LOQ	<LOQ	<LOQ
La 9/19	Atlantic cod	n.d.	n.d.	n.d.	n.d.	n.d.	<LOQ
La 10/19	Atlantic cod	n.d.	n.d.	n.d.	<LOQ	<LOQ	<LOQ
La 11/19	Atlantic cod	n.d.	n.d.	n.d.	<LOQ	<LOQ	<LOQ
La 13/19	Atlantic cod	n.d.	n.d.	n.d.	<LOQ	<LOQ	n.d.
La 14/19	Atlantic cod	n.d.	n.d.	n.d.	<LOQ	<LOQ	<LOQ
La 15/19	Atlantic cod	n.d.	n.d.	n.d.	<LOQ	<LOQ	<LOQ
La 103/19; La 106/19 La 108/19; La 109/19 La 110/19; La 113/19 La 122/19	European flounder	n.d.	n.d.	n.d.	n.d.	n.d.	n.d.
La 104/19	European flounder	n.d.	n.d.	n.d.	2.24	<LOQ	1.31
La 105/19	European flounder	n.d.	n.d.	n.d.	<LOQ	<LOQ	n.d.
La 115/19	European flounder	n.d.	n.d.	n.d.	<LOQ	<LOQ	n.d.
La 116/19	European flounder	n.d.	n.d.	n.d.	<LOQ	<LOQ	n.d.
La 102/19; La 112/19 La 120/19; La 121/19 La126/19; La 126/19	Lemon sole	n.d.	n.d.	n.d.	n.d.	n.d.	n.d.
La 124/19	Lemon sole	n.d.	<LOQ	<LOQ	4.63	<LOQ	<LOQ
La 114/19 La 117/19–119/19	European plaice	n.d.	n.d.	n.d.	n.d.	n.d.	n.d.
La 123/19	European plaice	n.d.	n.d.	n.d.	<LOQ	<LOQ	n.d.
La 107/19	European plaice	n.d.	n.d.	<LOQ	13.7	<LOQ	<LOQ

**Table 3:** Concentration range (expressed in ng/g fresh weight) and detection frequency (%) of each specific class of contaminant in the fillets of four demersal fish species caught at Siragrunnen and Lafjorden. n.d. = not detected; n.a. = not available because the contaminants were never detected. Data for European flounder in Siragrunnen were not available because no individual of this species was caught in the fishing area.

	Atlantic Cod		European Flounder		Lemon Sole		European Plaice	
	Concentration Range (ng/g fw)	Detection Frequency (%)	Concentration Range (ng/g fw)	Detection Frequency (%)	Concentration Range (ng/g fw)	Detection Frequency (%)	Concentration Range (ng/g fw)	Detection Frequency (%)
**Siragrunnen**								
ΣOCs	n.a.	n.d.			n.a.	n.d.	n.d.–4.5	75%
ΣOPs	n.a.	n.d.			n.a.	n.d.	n.a.	n.d.
ΣPCBs	n.d.–0.30	6%			n.a.	n.d.	n.a.	n.d.
ΣPAHs	n.a.	n.d.			n.a.	n.d.	n.a.	n.d.
ΣPBDEs	n.a.	n.d.			n.a.	n.d.	n.a.	n.d.
ΣPFASs	n.d.–0.26	6%			n.d.–2.34	40%	n.d.–0.12	25%
**Lafjorden**								
ΣOCs	n.d.–1.5	22%	n.d.–7.23	27%	n.d.–3	43%	n.d.–4.5	33%
ΣOPs	n.a.	n.d.	n.a.	n.d.	n.a.	n.d.	n.a.	n.d.
ΣPCBs	n.d.–0.15	66%	n.d.–3.7	36%	n.d.–5.23	14%	n.d.–14.15	33%
ΣPAHs	n.a.	n.d.	n.a.	n.d.	n.a.	n.d.	n.a.	n.d.
ΣPBDEs	n.a.	n.d.	n.a.	n.d.	n.a.	n.d.	n.a.	n.d.
ΣPFASs	n.d.–0.38	22%	n.d.–0.12	9%	n.d.–0.22	28%	n.a.	n.d.

**Table 4:** Levels of per- and polyfluoroalkyl substances (PFASs) measured in fillet of the four demersal fish species caught at Siragrunnen and Lafjorden. n.d. = not detected (i.e., <LOD = below the limit of detection. LOD values for all the PFASs are reported in Table S3. Levels are expressed as ng/g fresh weight. Perfluorobutanoic acid (PFBA), perfluoropentanoic acid (PFPeA), perfluorohexanoic acid (PFHxA), perfluorobutane sulphonic acid (PFBS), perfluoroheptanoic acid (PFHpA), perfluorooctanoic acid (PFOA), perfluorohexane sulphonate (PFHxS), perfluorononanoic acid (PFNA), perfluorodecanoic acid (PFDA), perfluorooctane sulfonic acid (PFOS), perfluorododecanoic acid (PFDoA), perfluoroundecanoic acid (PFUnDA), sodium perfluoro-1-decanesulfonate (PFDS), perfluorotridecanoic acid (PFTrDA), perfluorotetradecanoic acid (PFTeDA), perfluorohexadecanoic acid (PFHxDA), and perfluorooctadecanoic acid (PFODA).

Id	Species	PFBA	PFPeA	PFBS	PFHxA	PFHpA	PFHxS	PFOA	PFNA	PFOS	PFDA	PFUdA	PFDS	PFDoA	PFTrDA	PFTeDA	PFHxDA	PFODA
**Siragrunnen**																		
OS 1/19–10/19; OS 11/19–14/19	Atlantic cod	n.d.	n.d.	n.d.	n.d.	n.d.	n.d.	n.d.	n.d.	n.d.	n.d.	n.d.	n.d.	n.d.	n.d.	n.d.	n.d.	n.d.
OS 10/19	Atlantic cod	n.d.	n.d.	n.d.	n.d.	n.d.	n.d.	0.26	n.d.	n.d.	n.d.	n.d.	n.d.	n.d.	n.d.	n.d.	n.d.	n.d.
OS 100/19 OS 102/19 OS 104/19	Lemon sole	n.d.	n.d.	n.d.	n.d.	n.d.	n.d.	n.d.	n.d.	n.d.	n.d.	n.d.	n.d.	n.d.	n.d.	n.d.	n.d.	n.d.
OS 101/19	Lemon sole	n.d.	n.d.	n.d.	n.d.	n.d.	n.d.	0.66	n.d.	n.d.	n.d.	n.d.	n.d.	n.d.	n.d.	n.d.	n.d.	n.d.
OS 103/19	Lemon sole	n.d.	n.d.	n.d.	n.d.	n.d.	n.d.	2.34	n.d.	n.d.	n.d.	n.d.	n.d.	n.d.	n.d.	n.d.	n.d.	n.d.
OS 106/19	European plaice	n.d.	n.d.	n.d.	n.d.	n.d.	n.d.	0.12	n.d.	n.d.	n.d.	n.d.	n.d.	n.d.	n.d.	n.d.	n.d.	n.d.
OS 105/19 OS 107/19 OS 108/19	European plaice	n.d.	n.d.	n.d.	n.d.	n.d.	n.d.	n.d.	n.d.	n.d.	n.d.	n.d.	n.d.	n.d.	n.d.	n.d.	n.d.	n.d.
**Lafjorden**																		
La 1/19–6/19; La 8/19; La 9/19; La 11/19–14/19 La 16/19 La 17/19	Atlantic cod	n.d.	n.d.	n.d.	n.d.	n.d.	n.d.	n.d.	n.d.	n.d.	n.d.	n.d.	n.d.	n.d.	n.d.	n.d.	n.d.	n.d.
La 7/19	Atlantic cod	n.d.	n.d.	n.d.	n.d.	n.d.	n.d.	n.d.	n.d.	0.27	n.d.	n.d.	n.d.	n.d.	n.d.	n.d.	n.d.	n.d.
La 10/19	Atlantic cod	n.d.	n.d.	n.d.	n.d.	n.d.	n.d.	n.d.	n.d.	0.22	n.d.	n.d.	n.d.	n.d.	n.d.	n.d.	n.d.	n.d.
La 15/19	Atlantic cod	n.d.	n.d.	n.d.	n.d.	n.d.	n.d.	n.d.	n.d.	0.38	n.d.	n.d.	n.d.	n.d.	n.d.	n.d.	n.d.	n.d.
La 18/19	Atlantic cod	n.d.	n.d.	n.d.	n.d.	n.d.	n.d.	n.d.	n.d.	0.30	n.d.	n.d.	n.d.	n.d.	n.d.	n.d.	n.d.	n.d.
La 103/19–116/19	European flounder	n.d.	n.d.	n.d.	n.d.	n.d.	n.d.	n.d.	n.d.	n.d.	n.d.	n.d.	n.d.	n.d.	n.d.	n.d.	n.d.	n.d.
La 122/19	European flounder	n.d.	n.d.	n.d.	n.d.	n.d.	n.d.	n.d.	n.d.	0.12	n.d.	n.d.	n.d.	n.d.	n.d.	n.d.	n.d.	n.d.
La 102/19 La 112/19 La 120/19 La 125/19 La 126/19	Lemon sole	n.d.	n.d.	n.d.	n.d.	n.d.	n.d.	n.d.	n.d.	n.d.	n.d.	n.d.	n.d.	n.d.	n.d.	n.d.	n.d.	n.d.
La 121/19	Lemon sole	n.d.	n.d.	n.d.	n.d.	n.d.	n.d.	n.d.	n.d.	0.22	n.d.	n.d.	n.d.	n.d.	n.d.	n.d.	n.d.	n.d.
La 124/19	Lemon sole	n.d.	n.d.	n.d.	n.d.	n.d.	n.d.	n.d.	n.d.	0.20	n.d.	n.d.	n.d.	n.d.	n.d.	n.d.	n.d.	n.d.
La 117/19–119/19; La 107/19 La 114/19 La 123/19	European plaice	n.d.	n.d.	n.d.	n.d.	n.d.	n.d.	n.d.	n.d.	n.d.	n.d.	n.d.	n.d.	n.d.	n.d.	n.d.	n.d.	n.d.

## Supplementary Materials

The following are available online at https://www.mdpi.com/2304-8158/9/8/1108/s1, Table S1: Identification number, common and specific name, site of sampling and coordinates, total length, total weight and sex (M = male; F = female; n.s. = not sexed) of each single individual of the four demersal fish species caught at Siragrunnen in 2019, Table S2: Identification number, common and specific name, site of sampling and coordinates, total length, total weight and sex (M = male; F = female; n.s. = not sexed) of each single individual of the four demersal fish species caught at Lafjorden in 2019. Table S3: Limit of detection (LOD), limit of quantification (LOQ), repeatability (CV%) and recovery (%) for each investigated compound.
