# Supplementary material for: Legacy and Emerging Contaminants in Demersal Fish Species from Southern Norway and Implications for Food Safety
Source: Foods. 2020 Aug 12;9(8):1108. doi: 10.3390/foods9081108 (PMC7466181; doi:10.3390/foods9081108)
Supplement: Supplementary file 1 [file foods-09-01108-s001.zip › Table S1_R1.docx]

| **ID** | **Sampling date** | **Sampling site** | **Coordinates (WGS 84)** | **Common name** | **Binomial**  **nomenclature** | **Total length**  **(cm)** | **Body weight**  **(kg)** | **Sex** |
| --- | --- | --- | --- | --- | --- | --- | --- | --- |
| OS 1/19 | 20/03/2019 | Siragrunnen | 58°14.899’, 6°20.500’ | Atlantic cod | *Gadus morhua* | 64 | 2.50 | M |
| OS 2/19 | 20/03/2019 | Siragrunnen | 58°14.899’, 6°20.500’ | Atlantic cod | *Gadus morhua* | 55.5 | 1.48 | F |
| OS 3/19 | 20/03/2019 | Siragrunnen | 58°14.899’, 6°20.500’ | Atlantic cod | *Gadus morhua* | 49 | 1.12 | F |
| OS 4/19 | 20/03/2019 | Siragrunnen | 58°14.899’, 6°20.500’ | Atlantic cod | *Gadus morhua* | 57 | 1.90 | F |
| OS 5/19 | 20/03/2019 | Siragrunnen | 58°14.899’, 6°20.500’ | Atlantic cod | *Gadus morhua* | 59.5 | 2.16 | M |
| OS 6/19 | 20/03/2019 | Siragrunnen | 58°14.899’, 6°20.500’ | Atlantic cod | *Gadus morhua* | 71 | 4.00 | M |
| OS 7/19 | 26/03/2019 | Siragrunnen | 58°14.899’, 6°20.500’ | Atlantic cod | *Gadus morhua* | 86 | 4.76 | F |
| OS 8/19 | 26/03/2019 | Siragrunnen | 58°14.899’, 6°20.500’ | Atlantic cod | *Gadus morhua* | 51.5 | 1.44 | M |
| OS 9/19 | 26/03/2019 | Siragrunnen | 58°14.899’, 6°20.500’ | Atlantic cod | *Gadus morhua* | 64 | 2.46 | F |
| OS 10/19 | 26/03/2019 | Siragrunnen | 58°14.899’, 6°20.500’ | Atlantic cod | *Gadus morhua* | 65.5 | 2.76 | M |
| OS 11/19 | 26/03/2019 | Siragrunnen | 58°14.899’, 6°20.500’ | Atlantic cod | *Gadus morhua* | 49 | 1.00 | F |
| OS 12/19 | 26/03/2019 | Siragrunnen | 58°14.899’, 6°20.500’ | Atlantic cod | *Gadus morhua* | 48 | 0.90 | F |
| OS 13/19 | 26/03/2019 | Siragrunnen | 58°14.899’, 6°20.500’ | Atlantic cod | *Gadus morhua* | 45.5 | 0.96 | M |
| OS 14/19 | 17/02/2019 | Skageflua | 58°11.578’, 6°40.390’ | Atlantic cod | *Gadus morhua* | 55 | 1.95 | F |
| OS 100/19 | 26/03/2019 | Siragrunnen | 58°14.899’, 6°20.500’ | Lemon sole | *Microstomus kitt* | 33 | 0.39 | F |
| OS 101/19 | 26/03/2019 | Siragrunnen | 58°14.899’, 6°20.500’ | Lemon sole | *Microstomus kitt* | 33.5 | 0.40 | F |
| OS 102/19 | 26/03/2019 | Siragrunnen | 58°14.899’, 6°20.500’ | Lemon sole | *Microstomus kitt* | 35 | 0.50 | M |
| OS 103/19 | 26/03/2019 | Siragrunnen | 58°14.899’, 6°20.500’ | Lemon sole | *Microstomus kitt* | 33.5 | 0.39 | F |
| OS 104/19 | 26/03/2019 | Siragrunnen | 58°14.899’, 6°20.500’ | Lemon sole | *Microstomus kitt* | 32 | 0.34 | F |
| OS 105/19 | 26/03/2019 | Siragrunnen | 58°14.899’, 6°20.500’ | European Plaice | *Pleuronectes platessa* | 29.5 | 0.27 | M |
| OS 106/19 | 26/03/2019 | Siragrunnen | 58°14.899’, 6°20.500’ | European Plaice | *Pleuronectes platessa* | 34.5 | 0.35 | M |
| OS 107/19 | 26/03/2019 | Siragrunnen | 58°14.899’, 6°20.500’ | European Plaice | *Pleuronectes platessa* | 45.5 | 0.87 | F |
| OS 108/19 | 26/03/2019 | Siragrunnen | 58°14.899’, 6°20.500’ | European Plaice | *Pleuronectes platessa* | 44.5 | 0.95 | F |
|  |  |  |  |  |  |  |  |  |
|  |  |  |  |  |  |  |  |  |
