# Supplementary material for: Legacy and Emerging Contaminants in Demersal Fish Species from Southern Norway and Implications for Food Safety
Source: Foods. 2020 Aug 12;9(8):1108. doi: 10.3390/foods9081108 (PMC7466181; doi:10.3390/foods9081108)
Supplement: Supplementary file 1 [file foods-09-01108-s001.zip › Table S2_R1.docx]

| **ID** | **Sampling date** | **Location** | **Coordinates (WGS 84)** | **Common name** | **Binomial**  **nomenclature** | **Total length**  **(cm)** | **Body weight**  **(kg)** | **Sex** |
| --- | --- | --- | --- | --- | --- | --- | --- | --- |
| La 1/19 | 30/01/2019 | Raulibukta | 58°17.012’, 6°40.124’ | Atlantic cod | *Gadus morhua* | 65 | 3.01 | F |
| La 2/19 | 05/02/2019 | Raulibukta | 58°17.012’, 6°40.124’ | Atlantic cod | *Gadus morhua* | 56 | 1.78 | F |
| La 3/19 | 09/02/2019 | Lafjorden | 58°15.867’, 6°38.919’ | Atlantic cod | *Gadus morhua* | 76 | 4.20 | M |
| La 4/19 | 26/02/2019 | Raulibukta | 58°17.012’, 6°40.124’ | Atlantic cod | *Gadus morhua* | 55.5 | 1.50 | M |
| La 5/19 | 01/03/2019 | Grønnes | 58°17.202’, 6°39.731’ | Atlantic cod | *Gadus morhua* | 55.5 | 1.80 | F |
| La 6/19 | 11/03/2019 | Raulibukta | 58°17.012’, 6°40.124’ | Atlantic cod | *Gadus morhua* | 74 | 4.87 | F |
| La 7/19 | 11/03/2019 | Fjellse | 58°16.213’, 6°38.782’ | Atlantic cod | *Gadus morhua* | 60 | 2.01 | M |
| La 8/19 | 11/03/2019 | Raulibukta | 58°17.012’, 6°40.124’ | Atlantic cod | *Gadus morhua* | 46 | 0.92 | M |
| La 9/19 | 11/03/2019 | Raulibukta | 58°17.012’, 6°40.124’ | Atlantic cod | *Gadus morhua* | 40 | 0.70 | M |
| La 10/19 | 11/03/2019 | Grønnes | 58°17.202’, 6°39.731’ | Atlantic cod | *Gadus morhua* | 36 | 0.42 | F |
| La 11/19 | 12/03/2019 | Lafjorden | 58°15.867’, 6°38.919’ | Atlantic cod | *Gadus morhua* | 62 | 2.20 | M |
| La 12/19 | 12/03/2019 | Lafjorden | 58°15.867’, 6°38.919’ | Atlantic cod | *Gadus morhua* | 60 | 2.00 | F |
| La 13/19 | 12/03/2019 | Lafjorden | 58°15.867’, 6°38.919’ | Atlantic cod | *Gadus morhua* | 60 | 2.30 | M |
| La 14/19 | 12/03/2019 | Lafjorden | 58°15.867’, 6°38.919’ | Atlantic cod | *Gadus morhua* | 68 | 2.81 | M |
| La 15/19 | 12/03/2019 | Lafjorden | 58°15.867’, 6°38.919’ | Atlantic cod | *Gadus morhua* | 68 | 2.60 | F |
| La 16/19 | 26/03/2019 | Lafjorden | 58°15.867’, 6°38.919’ | Atlantic cod | *Gadus morhua* | 59 | 2.18 | F |
| La 17/19 | 26/03/2019 | Lafjorden | 58°15.867’, 6°38.919’ | Atlantic cod | *Gadus morhua* | 63.5 | 2.30 | F |
| La 18/19 | 26/03/2019 | Lafjorden | 58°15.867’, 6°38.919’ | Atlantic cod | *Gadus morhua* | 65.5 | 2.60 | F |
| La 102/19 | 04/02/2019 | Berrefjords | 58°15.759’, 6°27.972’ | European flounder | *Platichthys flesus* | 35 | 0.54 | M |
| La 103/19 | 05/02/2019 | Raulibukta | 58°17.012’, 6°40.124’ | European flounder | *Platichthys flesus* | 32.5 | 0.34 | M |
| La 104/19 | 05/02/2019 | Raulibukta | 58°17.012’, 6°40.124’ | European flounder | *Platichthys flesus* | 35 | 0.47 | F |
| La 105/19 | 05/02/2019 | Raulibukta | 58°17.012’, 6°40.124’ | European flounder | *Platichthys flesus* | 40.5 | 1.04 | F |
| La 106/19 | 05/02/2019 | Raulibukta | 58°17.012’, 6°40.124’ | European flounder | *Platichthys flesus* | 36 | 0.54 | M |
| La 107/19 | 06/02/2019 | Lafjorden | 58°15.867’, 6°38.919’ | European flounder | *Platichthys flesus* | 30.5 | 0.36 | M |
| La 108/19 | 07/02/2019 | Lafjorden | 58°15.867’, 6°38.919’ | European flounder | *Platichthys flesus* | 35.5 | 0.55 | F |
| La 109/19 | 07/02/2019 | Lafjorden | 58°15.867’, 6°38.919’ | European flounder | *Platichthys flesus* | 39 | 0.75 | F |
| La 110/19 | 07/02/2019 | Lafjorden | 58°15.867’, 6°38.919’ | European flounder | *Platichthys flesus* | 44 | 1.20 | F |
| La 112/19 | 26/02/2019 | Raulibukta | 58°17.012’, 6°40.124’ | European flounder | *Platichthys flesus* | 35 | 0.54 | F |
| La 113/19 | 26/02/2019 | Raulibukta | 58°17.012’, 6°40.124’ | European flounder | *Platichthys flesus* | 44 | 0.89 | M |
| La 114/19 | 26/02/2019 | Raulibukta | 58°17.012’, 6°40.124’ | European Plaice | *Pleuronectes platessa* | 47 | 1.25 | F |
| La 115/19 | 11/03/2019 | Lafjorden | 58°15.867’, 6°38.919’ | European Plaice | *Pleuronectes platessa* | 34 | 0.33 | M |
| La 116/19 | 11/03/2019 | Lafjorden | 58°15.867’, 6°38.919’ | European Plaice | *Pleuronectes platessa* | 40 | 0.64 | F |
| La 117/19 | 11/03/2019 | Lafjorden | 58°15.867’, 6°38.919’ | European Plaice | *Pleuronectes platessa* | 38 | 0.62 | F |
| La 118/19 | 11/03/2019 | Lafjorden | 58°15.867’, 6°38.919’ | European Plaice | *Pleuronectes platessa* | 33 | 0.39 | F |
| La 119/19 | 11/03/2019 | Lafjorden | 58°15.867’, 6°38.919’ | European Plaice | *Pleuronectes platessa* | 33.5 | 0.39 | M |
| La 120/19 | 11/03/2019 | Lafjorden | 58°15.867’, 6°38.919’ | Lemon sole | *Microstomus kitt* | 36.5 | 0.51 | M |
| La 121/19 | 11/03/2019 | Lafjorden | 58°15.867’, 6°38.919’ | Lemon sole | *Microstomus kitt* | 37 | 0.40 | n.s. |
| La 122/19 | 12/03/2019 | Lafjorden | 58°15.867’, 6°38.919’ | Lemon sole | *Microstomus kitt* | 36 | 0.45 | n.s. |
| La 123/19 | 26/03/2019 | Lafjorden | 58°15.867’, 6°38.919’ | Lemon sole | *Microstomus kitt* | 31 | 0.34 | M |
| La 124/19 | 26/03/2019 | Lafjorden | 58°15.867’, 6°38.919’ | Lemon sole | *Microstomus kitt* | 35 | 0.58 | F |
| La 125/19 | 26/03/2019 | Lafjorden | 58°15.867’, 6°38.919’ | Lemon sole | *Microstomus kitt* | 34 | 0.39 | M |
| La 126/19 | 26/03/2019 | Lafjorden | 58°15.867’, 6°38.919’ | Lemon sole | *Microstomus kitt* | 35 | 0.44 | M |
