# Supplementary material for: Legacy and Emerging Contaminants in Demersal Fish Species from Southern Norway and Implications for Food Safety
Source: Foods. 2020 Aug 12;9(8):1108. doi: 10.3390/foods9081108 (PMC7466181; doi:10.3390/foods9081108)
Supplement: Supplementary file 1 [file foods-09-01108-s001.zip › Table S3_R1.docx]

| **Compound** | **LOD** | **LOQ** | **CV** | **Recovery** |
| --- | --- | --- | --- | --- |
|  | **(ng g^-1^)** | **(ng g^-1^)** | **%** | **%** |
| PCB 28 | 0.15 | 0.50 | 11 | 85 |
| PCB 52 | 0.15 | 0.50 | 9 | 87 |
| PCB 101 | 0.15 | 0.50 | 9 | 83 |
| PCB 138 | 0.15 | 0.50 | 12 | 97 |
| PCB 153 | 0.15 | 0.50 | 12 | 85 |
| PCB 180 | 0.15 | 0.50 | 10 | 88 |
| PBDE 28 | 0.15 | 0.50 | 2 | 93 |
| PBDE 33 | 0.15 | 0.50 | 3 | 79 |
| PBDE 47 | 0.15 | 0.50 | 9 | 94 |
| PBDE 99 | 0.15 | 0.50 | 7 | 81 |
| PBDE 100 | 0.15 | 0.50 | 11 | 80 |
| PBDE 153 | 0.15 | 0.50 | 7 | 70 |
| PBDE 154 | 0.15 | 0.50 | 9 | 84 |
| α-HCH | 1.50 | 5.00 | 18 | 119 |
| β-BHC | 1.50 | 5.00 | 20 | 120 |
| Hexachlorbenzene | 1.50 | 5.00 | 16 | 100 |
| Lindane | 1.50 | 5.00 | 20 | 116 |
| Heptachlor | 1.50 | 5.00 | 20 | 120 |
| Aldrin | 1.50 | 5.00 | 12 | 89 |
| Heptachlor epoxide | 1.50 | 5.00 | 10 | 93 |
| Trans chlordane | 1.50 | 5.00 | 12 | 94 |
| Endosulfan I | 1.50 | 5.00 | 12 | 95 |
| Endosulfan II | 1.50 | 5.00 | 10 | 84 |
| p,p'- DDE | 1.50 | 5.00 | 16 | 90 |
| Endosulfan Sulfate | 1.50 | 5.00 | 14 | 75 |
| Endrin | 1.50 | 5.00 | 10 | 120 |
| o,p'-DDT | 1.50 | 5.00 | 20 | 120 |
| p,p'- DDD | 1.50 | 5.00 | 3 | 102 |
| Chrysene | 0.15 | 0.50 | 3 | 82 |
| Antracene | 0.15 | 0.50 | 6 | 75 |
| Benzofluoranthene | 0.15 | 0.50 | 3 | 75 |
| Benzopyrene | 0.15 | 0.50 | 2 | 77 |
|  | **(pg g^-1^)** | **(pg g^-1^)** |  |  |
| PFBA | 1.50 | 5.00 | 7 | 82 |
| PFPeA | 3.00 | 10.00 | 10 | 114 |
| PFBS | 3.00 | 10.00 | 11 | 102 |
| PFHxA | 6.01 | 20.00 | 6 | 110 |
| PFHpA | 1.50 | 5.00 | 5 | 112 |
| PFHxS | 4.50 | 15.00 | 9 | 103 |
| PFOA | 1.50 | 5.00 | 5 | 113 |
| PFNA | 1.50 | 5.00 | 10 | 95 |
| PFOS | 1.50 | 5.00 | 12 | 83 |
| PFDA | 6.01 | 20.00 | 13 | 84 |
| PFUdA | 6.01 | 20.00 | 6 | 85 |
| PFDS | 6.01 | 20.00 | 8 | 83 |
| PFDoA | 6.01 | 20.00 | 8 | 89 |
| PFTrDA | 4.50 | 15.00 | 8 | 87 |
| PFTeDA | 1.50 | 5.00 | 15 | 91 |
| PFHxDA | 1.50 | 5.00 | 18 | 85 |
| PFODA | 1.50 | 5.00 | 18 | 84 |
